# Validation of the Indian (Hindi) version of the life-space assessment scale among community-dwelling older adults: a multicentric cross-sectional study

**DOI:** 10.1186/s12877-024-05072-4

**Published:** 2024-06-06

**Authors:** Durgesh Prasad Sahoo, Soumya Swaroop Sahoo, Santosh Kumar Nirala, Rama Shankar Rath, Neeraj Agarwal, Meely Panda, Rakesh Kakkar, Sanjay Pandey, C M Singh, Hari Shanker Joshi, Bhola Nath

**Affiliations:** 1 Dept. of Community Medicine and Family Medicine, All India Institute of Medical Sciences, Bibinagar, Hyderabad India; 2 Dept. of Community and Family Medicine, All India Institute of Medical Sciences, Bathinda, India; 3 Dept. of Community and Family Medicine, All India Institute of Medical Sciences, Patna, India; 4 Dept. of Community Medicine and Family Medicine, All India Institute of Medical Sciences, Gorakhpur, India; 5 Dept. of Community Medicine, All India Institute of Medical Sciences, Raebareli, India

**Keywords:** Life-space assessment, Older adults, Validity, Reliability, Mobility, Hindi version

## Abstract

**Background:**

The Life-Space Assessment (LSA) is an instrument that measures mobility in older adults as they reach different areas, defined as life-spaces extending from home to beyond towns or regions. The purpose of the study was to develop the Hindi version of the LSA (LSA- H) and to investigate the validity and reliability of the Hindi version as well as its cultural adaptation.

**Methods:**

A cross-sectional study of two hundred forty-five older adults participated in the study from four different study practice areas. Following forward backwards translation, the LSA-H was developed, and the scores were correlated with those of the Activities-Specific Balance Confidence Scale Hindi (ABC- H), the Physical Health Subscale of the WHO-BREF Questionnaire and the Geriatric Depression Scale: Short Form Hindi (GDS-SFH) to test the criterion and concurrent validity.

**Results:**

The mean score and standard deviation of the LSA-H questionnaire were 56.53 ± 35.99, those of the Physical Health Subscale of the WHO-BREF instrument were 18.54 ± 7.87, those of the GDS-SFH questionnaire were 6.95 ± 4.21 and those of the ABC- H questionnaire were 54.40 ± 28.96. The Pearson correlation coefficient (r) between the LSA-H score and ABC-H score was 0.707 (*p* value < 0.0001), that between the LSA-H score and the Physical Health Subscale of the WHO-BREF was 0.766 (*p* value < 0.0001), and that between the LSA-H score and GDS-SFG score was − 0.674 (*p* value < 0.0001).

**Conclusion:**

This study demonstrated that the Hindi version of the LSA is a valid and reliable instrument for assessing living space among older adults in the Hindi language in an Indian population. Furthermore, the LSA-H was significantly correlated with other health assessment tools in terms of functional mobility, general health status and mental well-being.

## Introduction

Globally, the geriatric population (60 years and above) will increase from 12 to 22% between 2015 and 2050 [1]. India is undergoing a demographic transition, and the geriatric population is expected to double to 20.8% in 2050 from 10.5% in 2000. India falls under the United Nations’ definition of ‘ageing’ countries (a country is defined as ‘ageing’ when the percentage of the population aged ≥ 60 years reaches 7%) and is a conspicuous example of a context in which morbidity and mortality patterns are changing rapidly [2]. Healthy aging is defined as “the process of developing and maintaining the functional ability that enables wellbeing in older age” [3]. Mobility is one of the key hallmarks of functional aging [4]. Mobility is defined as ‘the ability to move oneself (either independently or by using assistive devices or transportation) within environments that expand from one’s home to the neighborhood and regions beyond [5]. Decreased mobility among older adults has serious consequences for physical function, mental health, social relationships and quality of life [6–9].

Multiple mobility assessment scales, instruments, questionnaires such as activities of daily living (ADLs), instrumental activities of daily living (IADLs), functional status questionnaire (FSQ), Physical Mobility Scale, 36-Item Short Form Survey (SF-36), Elderly Mobility Scale (EMS), Hierarchical Assessment of Balance and Mobility (HABAM) and Physical Performance Mobility Examination (PPME) are available that target specific activities and provide information regarding the motor function and coordination for mobility. However, they do not consider the interaction of mobility with the living environment [10–17].

The Life-Space Assessment (LSA) is an instrument that measures mobility in older adults by assessing mobility in different areas; this tool is defined as life spaces extending from home to beyond towns or regions and was developed by Baker et al. as part of the Study of Aging Life-Space Assessment (LSA) by The University of Alabama at Birmingham (UAB) for community-dwelling older adults [18, 19]. The LSA has been translated into multiple languages such as Spanish, Swedish, Danish and Chinese, for adaptability and use. However, no study has validated the psychometric property of the LSA version in the Hindi language [20–23]. Among the 121 languages spoken in India, according to the 2011 census, most (43.63%) of the Indian population speaks the Hindi language, which is recognized as one of the official languages across the country [24]. Therefore, the purpose of this study was to validate the Hindi version of the LSA questionnaire (LSA- H) and to investigate the validity and reliability of its Hindi version as well as its cultural adaptation.

## Methods

This community-based cross-sectional study was conducted in the field practice areas of four Hindi language-speaking Indian states where Institutes of National Importance (INIs) are located. Hindi is one of the most common languages spoken and used in India. The four states are Uttar Pradesh, Bihar, Punjab and Telangana, and the community field practice areas of the Department of Community & Family Medicine departments of All India Institute of Medical Sciences (AIIMS) situated at Gorakhpur, Patna, Bathinda and Hyderabad were selected as the study sites. The study participants were older adults aged more than 60 years residing in the community, and data were collected between May 2022 and May 2023. The sample size was calculated using the free software for calculation of sample size available at https://wnarifin.github.io/ssc/ssalpha.html. using minimum acceptable Cronbach’s alpha of 0.7, with an expected Cronbach’s alpha of 0.8 at the 1% significance level and 90% power with 15 items and a 15% drop out rate as 231 [25]. Therefore, we collected data from at least 60 community-dwelling older adults from the field practice area of each of the health centers. Individuals were approached by home visits; consent was obtained, and they were interviewed for the life space assessment using the available study tool. The number of blocks in each of the districts where AIIMS is located was determined for the four states. One block was selected randomly, simple random sampling was used to select the houses, and all individuals aged more than 60 years were interviewed based on our inclusion criteria. The list of older adults was collected from the community health workers in that area and numbered. Only one older adult from each eligible household was included in the study sample. Older adults were selected randomly from the block by a simple random sampling method.

The inclusion criteria were age ≥ 60 years, residing in the community for at least one year, having a normal cognitive function score ≥ 24 according to the Hindi version of the Mini-Mental State Questionnaire (H-MMSE) and fluency in the Hindi language [26]. Participants with major psychiatric disorders (e.g., schizophrenia, major depressive disorder, and delirium), acute respiratory/ circulatory disorders such as chronic obstructive pulmonary disease, acute myocardial infarction, functional movement disorders such as parkinsonism or cerebrovascular disease with sequelae etc., and any orthopedic injury affecting gait during the 2-week period before the survey were excluded from the study.

The data collection team included residents and field assistants who were well trained regarding the standard procedures of the interviews and were well oriented regarding various skills and techniques of data collection, such as communication skills and interviewing. The study was approved by the Ethics Committee of all four INIs, due permission for the use of the tools and informed written consent were obtained from the participants before the study was conducted.

## Study tools

### The Life-space assessment (LSA): (18)

The LSA is an interview-based questionnaire that is freely available in the public domain. It consists of five life space levels and addresses activities during the past 4 weeks. The five levels are other rooms within the home (Life space Level-1), the area outside the home (Life space Level-2), the neighborhood (Life space Level-3), the outside of neighborhood (Life space Level-4) and the place outside the town (Life space Level-5). For each life space level, scores are calculated by multiplying the life space level by frequency (less than 1/week = 1, 1–3 times/week = 2, 4–6 times/week = 3 and daily = 4) and level of independence (1 = personal assistance, 1.5 = equipment only, 2 = no equipment or personal assistance). The total scores were added to yield one composite score ranging from 0 to 120, with 0 representing no mobility and 120 representing the highest mobility.

### Cross cultural adaptation and translation to the Hindi version (LSA-H)

The Hindi version of the LSA instrument was developed based on the guidelines and techniques recommended by Gjersing L et al. [27]. The translation was performed in a stepwise manner. The initial translation of the original instrument was performed by two bilingual translators who were fluent in both the English and Hindi languages. A synthesized translated version of the instrument was developed by the investigators and translators. Back translation was performed by two new bilingual translators who were fluent in the English language and had a good understanding of the Hindi language. The synthesized back-translated version of the instrument was developed by the authors and back-translators. An expert committee consisting of specialists from Orthopedics, General Medicine and two independent translators reviewed the original instrument, the back-translated instrument and the Hindi version. The expert committee recommended a few minor changes on the basis of cross-cultural adaptation such as Life Space Level 2 perch, hallways that are uncommon in the country, were replaced by courtyards/gardens. Similarly, at Life space Levels 4 and 5, towns are replaced by villages/towns. Finally, the Hindi version was pretested with 20 community-dwelling older adults and after a few corrections in the grammatical error, the final Hindi version was developed.

### Activities-specific balance confidence scale Hindi (ABC- H)

The activity-specific balance confidence (ABC) scale is a self-report questionnaire to measure confidence in performing various ambulatory activities without losing balance; this scale was developed and published in English by Powell and Myers in 1995 [28]. This instrument consists of 16-items, and each item has a score of 0, which represents no confidence, and a score of 100, which represents complete confidence in performing specific activities. The overall score of the instrument is calculated by summing all the individual item scores and then dividing by the total number of items. The validated Hindi version of the ABC scale (ABC-H) has a Cronbach’s α value of 0.97 − 0.88 [29]. A Previous study by Neida et al. revealed a positive correlation between the ABC scale score and LSA scale score [30]. Therefore, the Hindi version of the ABC scale was used to assess the criterion validity of the Hindi version of the LSA.

### Physical health subscale of the WHO-BREF questionnaire

The physical health subscale of the WHO-BREF Questionnaire comprises 7 items; activities of daily living, dependence on medicinal substances and medical aids, energy and fatigue, mobility, pain and discomfort, sleep and rest and work capacity. The questions are scored using a five-point scale in which 1 = not at all/very dissatisfied, 2 = a little/dissatisfied, 3 = a moderate amount/moderately/neither satisfied or satisfied, 4 = very much/mostly/satisfied, and 5 = an extreme amount/completely/very satisfied. Of the 7 items, two (dependence on medicinal substances and medical aids and pain and discomfort) were reverse scored. The total score ranges from 5 to 35. The validated Hindi version is available in the public domain [31]. A previous study conducted by Rantakokko M et al. showed that life space mobility is proportional to quality of life [32]. The Hindi version of the WHO-BREF, which is also used to assess quality of life, was also used to assess the criterion validity of the Hindi version of the LSA.

### Geriatric depression scale: short form Hindi (GDS-SFH)

The GDS-SF assesses depressive symptoms in the past week and is a smaller version of the original GDS. It has 15 items with yes or no answer. Questions 1, 5, 7, 11 and 13 with answer numbers were given 1 point, while the remaining questions with yes were given 1 point. The score ranges from 0 to 15. A score greater than or equal to 5 was considered to indicate depression [33, 34]. The Hindi version is available from Ganguly M et al. and has internal consistency and a factor structure comparable to that of the original English language version [35]. Permission was given from the author to use the Hindi version. A study in which the Chinese version of the LSA was validated by Tseng YC et al. revealed a negative correlation with the 10-item Center for Epidemiological Studies Depression Scale (CESD-10) [21]. Therefore, the Hindi version of GDS-SF was used to assess content validity with hypothesis of LSA-H score may be negatively correlate with the GDS-SFH score.

### Sociodemographic factors

Sociodemographic factors, i.e., age, sex, education, marital status, occupation, per capita income, living arrangements, and the presence of comorbidities were assessed using a standardized questionnaire. Occupation status was classified according to the International Standard Classification of Occupations (ISCO-08), per capita income was classified according to the socioeconomic classification of the BG Prasad, and educational status was classified according to the Indian Standard Classification of Education [36–38].

### Statistical analysis

The validity of the LSA-H was assessed by content validity, criterion validity and construct validity and reliability were assessed by test- retest reliability [39]. The assessment of content validity, criterion validity, construct validity, and reliability adhered to the Consensus-based Standards for the Selection of Health Measurement Instruments (COSMIN) checklist [40]. 

### Content validity

Both the item-level content validity index (I-CVI) and scale level content validity index (S-CVI) were assessed for content validation. A panel of experts (6 in numbers) consisting of two faculty members in the field of General Medicine, two faculty from Public Health, and one from the Dept. of Orthopedics and one faculty member from the Department of Physical Medicine and Rehabilitation assessed the CVI using a four-point scale based on the degree of relevance (1 = the item is not relevant to the measured domain, 2 = the item is somewhat relevant to the measured domain, 3 = the item is quite relevant to the measured domain and 4 = the item is highly relevant to the measured domain) through a non-face-to-face approach. I-CVI and S-CVI values greater than 0.83 were considered as acceptable [41].

### Criterion validity

Criterion validity refers to how well scores on one measure (i.e., a predictor) correlate with scores on another measure of interest (i.e., the criterion) [39]. The LSA score is associated with activities of daily living, mobility and balance and gait; the WHO-BREF questionnaire and ABC-H questionnaire tool were used for assessing criterion validity [42]. The Pearson correlation coefficient was calculated between the LSA-H score and the physical health subscale scores of the WHO-BREF questionnaire and ABC-H questionnaire.

### Construct validity (hypotheses-testing)

Construct validity refers to the extent to which a measure of a construct adequately expresses the desired concept (i.e., it is free of measurement errors) and has been demonstrated to be an integral part of the research [43]. LSA scores are inversely associated with depression among older adults. The 15-item Geriatric Depression Scale- Short Form Hindi tool was used to assess construct validity [44]. The Pearson correlation coefficient was calculated between the LSA-H and the 15-item Geriatric Depression Scale- Short Form Hindi, with the hypothesis that the LSA-H score is negatively associated with the GDS- SFH score.

### Test- retest reliability

Test-retest stability refers to the stability of an instrument over time from one measurement session to another measurement session using the same evaluator, same scale and same subjects [39]. However, neither the evaluator nor the subjects were aware of the initial test results during the re-test, nor did they receive feedback on the results. The intraclass correlation coefficient (ICC) of the LSA-H was calculated to assess test- retest reliability. An ICC between 0.75 and 0.9 indicated good reliability, and values greater than 0.9 indicated excellent reliability [45]. The sample size for the test–retest analysis was calculated using the free software available at https://wnarifin.github.io/ssc/ssicc.html. using Intraclass Correlation Coefficient (ICC) with an expected ICC of 0.88, with a minimum acceptable reliability (ICC) of 0.6 with a significance level of 0.05, a power of 90%, two raters per subject and an expected dropout of 10% as 27. Therefore, the test- retest analysis was estimated by reassessing 30 subjects with an interval of one week between tests [21]. 

**Fig. 1:**
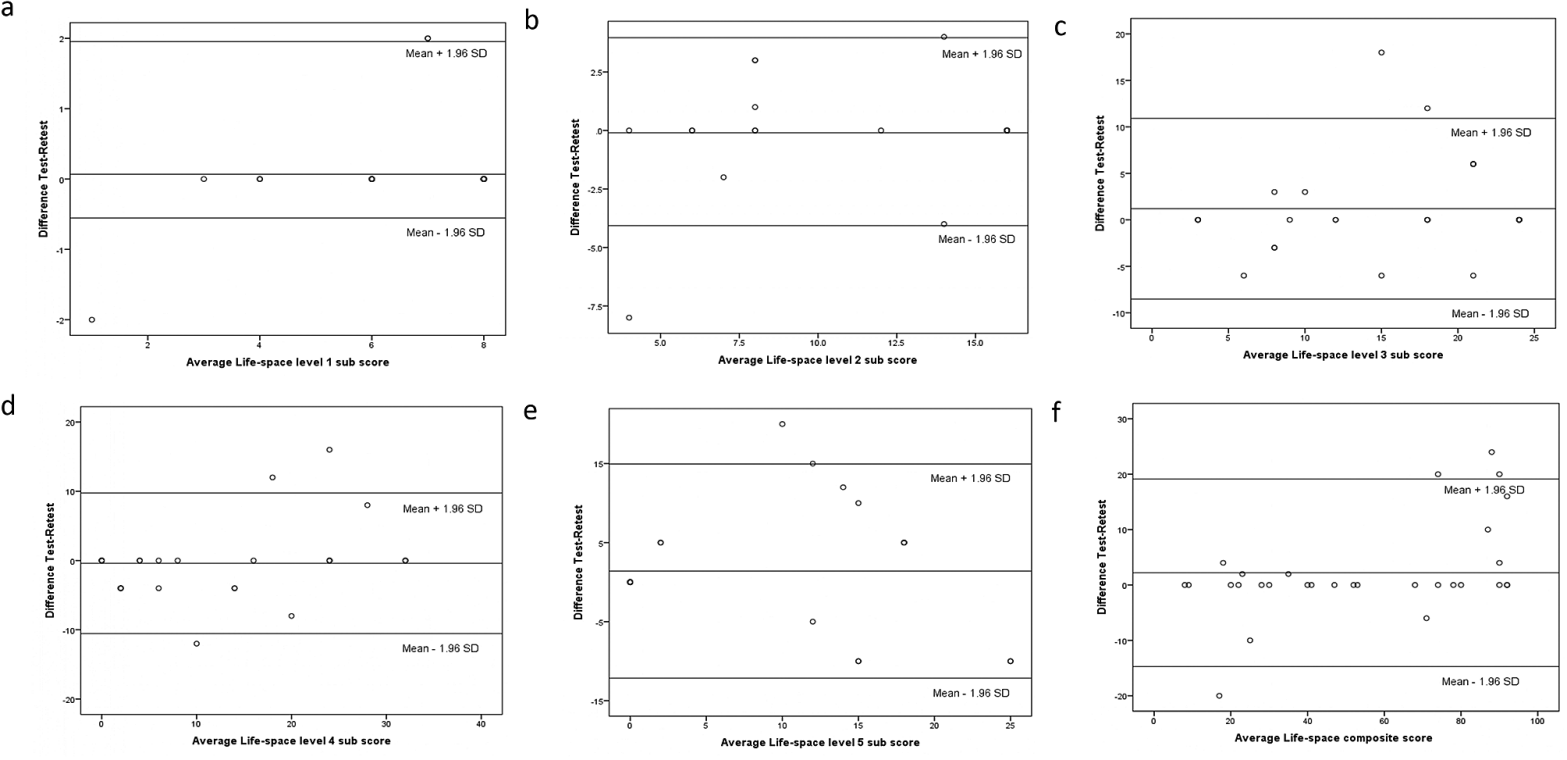
Bland–Altman showing the LSA Hindi questionnaire score agreement between test and retest in older adults for (**a**) Life-space level 1 sub score, (**b**) Life-space level 2 sub score, (**c**) Life-space level 3 sub score, (**d**) Life-space level 4 sub score, (**e**) Life-space level 5 sub score, (**f**) Life space composite score (*n* = 30)

The data were entered into Microsoft Excel and analysed using SPSS version 20.0 (IBM Corp., USA). There were no missing data and the data analysed were limited to those where complete data were available from the participants. The normality of the data was assessed by generating Q-Q plot, as were the skewness and kurtosis. Skewness and kurtosis values less than ± 1 were considered normally distributed. Categorical data are presented as frequencies and percentages, and the quantitative data are presented as the means, standard deviations (SD), medians and interquartile range (IQR). The criterion and construct validities were generated by calculating Pearson’s coefficients, and the test- retest reliability was assessed with a one-week interval and expressed as an intraclass correlation coefficient (ICC). The type of ICC used was one way random effects, absolute agreement, single rater/measurement ICC (1,1). The standard error of measurement (SEM) was calculated using the formula of SEM = SD_difference_ / √2 where SD _difference_ represents the standard error of the difference between test and retest score. The SEM% was computed using the formula of SEM/maximum score of the life space level expressed as percentage. The SEM% value less than 10% of the total sub score or composite score were considered as good, 10 to 20% as acceptable and more than 20% were regarded as doubtful. The Bland – Altman analysis was plotted to evaluate a bias between the mean differences, and to estimate an agreement interval, within which 95% of the differences of the retest score, compared to the test score, fall in older adults. The upper limit of agreement (Upper LOA) and the lower limit of agreement (Lower LOA) were calculated by mean _difference_ ± 1.96 SD _difference_. (Fig. 1) Cronbach’s alpha was utilized to evaluate internal consistency followed by tests of “scale if item deleted” with the removal of one item at the time of the questionnaire. A *p* value less than 0.05 was considered to indicate statistical significance.

## Results

A total of 245 participants were included in the present study. Of those, 60 patients were from the Hyderabad, Gorakhpur, and Patna centers, while 65 patients were from the Bathinda Center. The mean age of the study participants was 67.76 ± 7.61 years, and the majority of them were male (58.78%), illiterate (41.63%) or married (84.49%). Most of the study participants were homemakers (25.71%), followed by those involved in skilled agricultural, forestry and fishery work (20.00%) and those in elementary occupations (15.51%). The majority (51.02%) of the study participants had incomes less than a total of Rs. 2544.00 Indian Rupees (< 1272; 39.59%, 1272–2543; 11.43%) and 43.26% were living with their spouse only. The most common associated comorbidity was hypertension (40.82%), followed by diabetes mellitus (26.94%) (Table 1).

**Table 1:** Socio-demographic variables of the study participants

Sociodemographic variables (*n* = 245)		Numbers (*n*)	Percent	Mean ± SD
Age in years				67.76 ± 7.61
Gender	Female	101	41.22	
	Male	144	58.78	
Education	Illiterate	102	41.63	
	Primary	48	19.59	
	Upper Primary	33	13.47	
	Secondary	19	7.75	
	Senior Secondary	12	4.9	
	Graduate	21	8.58	
	Post Graduate and above	10	4.08	
Marital status	Married	207	84.49	
	Single	4	1.63	
	Widowed	34	13.88	
Occupation	Professionals	6	2.45	
	Technicians and associate professionals	30	12.25	
	Clerical support workers	6	2.45	
	Service and sales workers	16	6.53	
	Skilled agricultural, forestry and fishery workers	49	20.00	
	Elementary occupations	38	15.51	
	Home maker	63	25.71	
	None	37	15.10	
Per capita Income (in Indian Rupees)	≥ 8480	57	23.27	
	4240–8479	39	15.91	
	2544–4239	24	9.80	
	1272–2543	28	11.43	
	< 1272	97	39.59	
Living arrangement	Living with spouse only	106	43.26	
	Living with children only	70	28.57	
	Living with someone	62	25.31	
	Living alone	7	2.86	
Associated comorbidities	Hypertension	100	40.82	
	Diabetes Mellitus	66	26.94	
	Cardiac Disease	27	11.02	
	Cancer	5	2.04	
	Asthma	3	1.22	

The mean score and standard deviation of the LSA- H questionnaire score were 56.53 ± 35.99, the physical health subscale score of the WHO-BREF instrument was 18.54 ± 7.87, the GDS-SFH score was 6.95 ± 4.21, and the ABC-H score was 54.40 ± 28.96 (Table 2).

**Table 2:** Descriptive analysis of the study tools

Measures (*n* = 245)	Mean ± SD	Median (IQR)	Minimum	Maximum
LSA- H	56.53 ± 35.99	56.00 (24.00–90.00)	0	120
Physical health subscale of WHO-BREF	18.54 ± 7.87	20.00 (12.00–25.00)	7	35
GDS- SFH	6.95 ± 4.21	7.00 (3.00–11.00)	0	15
ABC- H	54.40 ± 28.96	58.12 (31.87–77.18)	0	100

### Content validity

The item-level content validity index (I-CVI) was 0.967, and the scale-level content validity index (S-CVI) was 0.965 for the LSA- H instrument for the panel of six experts.

### Criterion validity

**Fig. 2:**
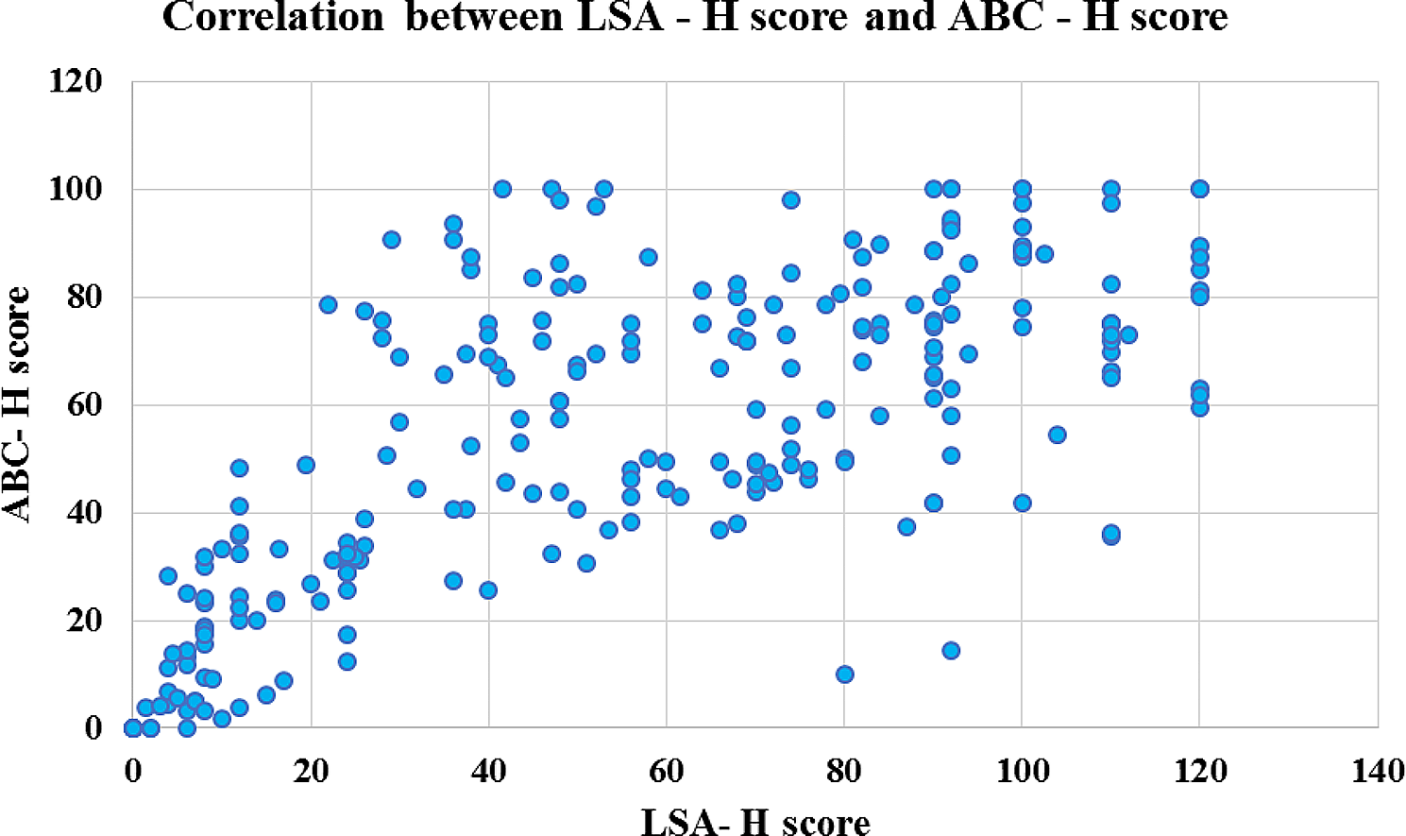
Correlations between the LSA-H score and the ABC-H score

**Fig. 3:**
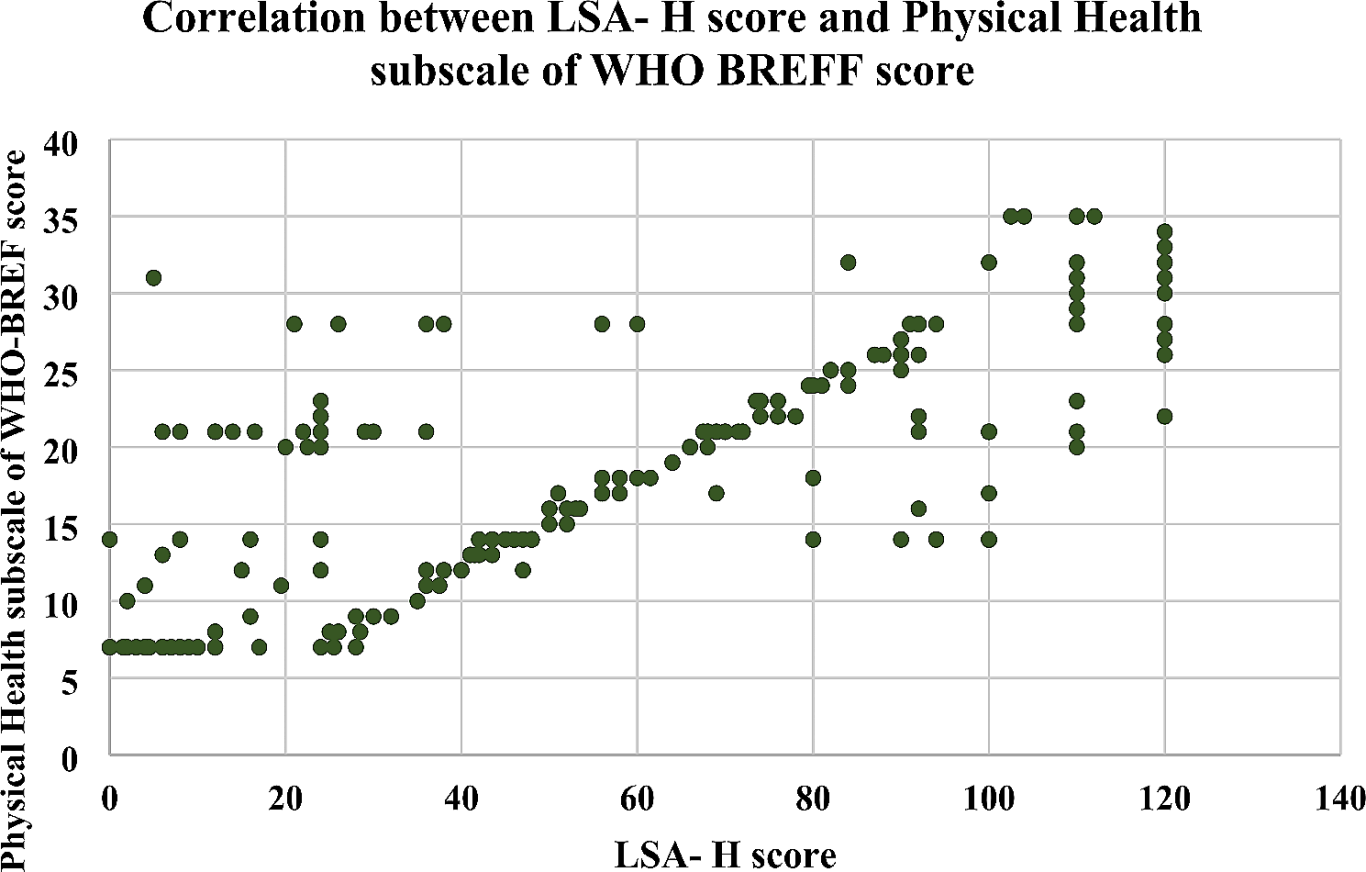
Correlations between the LSA-H score and scores on the Physical Health Subscale of the WHO-BREF instrument

The Pearson correlation coefficient (r) between the LSA-H score and the ABC-H score was 0.707 (*p* value < 0.0001), and that between the LSA-H score and the physical health subscale of the WHO-BREF was 0.766 (*p* value < 0.0001). These findings showed statistically significant positive correlations between the two scores and the LSA-H instrument, leading to acceptable criterion validity (Table 3; Figs. 2 and 3).

**Table 3:** Correlations of the LSA-H score with the ABC-H score, the physical health subscale of the WHO-BREF score and the GDS-SFH score

Measure	Mean ± SD	Pearson Correlation (*r*)	*p* value
ABC-H	54.40 ± 28.96	0.707	0.0001
Physical health subscale of WHO-BREF	18.54 ± 7.87	0.766	0.0001
GDS-SFH	6.95 ± 4.21	-0.674	0.0001

### Construct validity

**Fig. 4:**
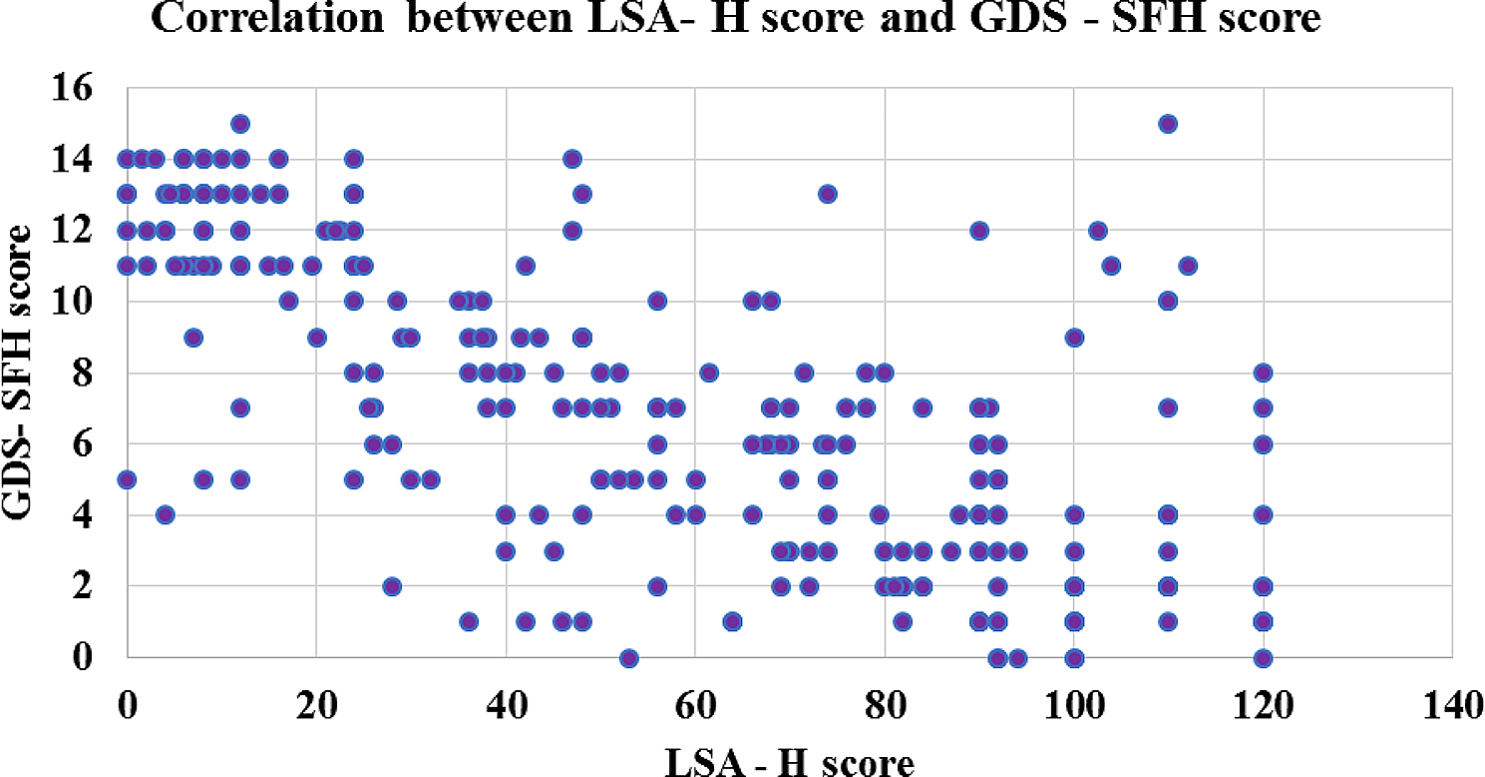
Correlations between the LSA-H score and GDS-SFH score

The Pearson correlation coefficient (r) between the LSA-H score and GDS-SFH score was − 0.674 (*p* value < 0.0001), indicating that mobility was negatively associated with depression and hence led to acceptable construct validity (Table 3; Fig. 4).

### Test- retest reliability

The test-retest reliability was estimated by reassessing 30 subjects after an interval of one week. The mean LSA composite test and retest scores were 55.53 ± 32.49 and 53.37 ± 27.97, respectively, with an ICC of 0.868, indicating good test-retest reliability. The ICC of the Life-space level 1 subscore indicated excellent reliability (ICC = 0.948), while the Life-space level 2–5 subscore showed good test-retest reliability (ICC = 0.88, 0.738, 0.871 and 0.843, respectively). The SEM % of the life space level 1 sub score, life space level 2 sub score and life space composite score were all less than 10% indicating a good reliability. Conversely, the SEM% for the life space level 3 sub score, life space level 4 sub score & life space level 5 sub score fell between 10 and 20%, which is considered as acceptable. (Table 4). The internal consistency as measured by Cronbach’s alpha was deemed appropriate with a value of 0.924 (0.912–0.928). This indicates high reliability and consistency among the items within the questionnaire.

**Table 4:** Standard error of measurement, upper and lower limit of agreement by Bland- Altman plot & intraclass correlation coefficients (ICCs) of the Hindi version of the life-space assessment

*n* = 30	Assessment	Mean ± SD	SEM	SEM%	Upper LOA- Lower LOA	ICC	95% CI (ICC)
Life-space level 1 sub score	1st assessment	6.8 ± 1.85	0.453	5.66%	1.954, -0.554	0.948	0.804, 0.952
	2nd assessment	6.73 ± 1.83					
Life-space level 2 sub score	1st assessment	12.57 ± 4.72	1.431	8.94%	3.865, -4.065	0.88	0.746, 0.943
	2nd assessment	12.67 ± 4.36					
Life-space level 3 sub score	1st assessment	15.90 ± 8.34	3.506	14.60%	10.917, -8.517	0.738	0.553, 0.875
	2nd assessment	14.70 ± 7.52					
Life-space level 4 sub score	1st assessment	12.20 ± 12.49	3.666	11.45%	9.761, -10.561	0.871	0.728, 0.939
	2nd assessment	12.60 ± 10.90					
Life-space level 5 sub score	1st assessment	8.00 ± 9.15	4.888	12.22%	14.946, -12.146	0.843	0.674, 0.925
	2nd assessment	6.60 ± 9.48					
Life-space composite score	1st assessment	55.53 ± 32.49	6.107	5.08%	19.127, -14.727	0.868	0.723, 0.937
	2nd assessment	53.37 ± 27.97					

## Discussion

In the present study, we developed an Indian version of the LSA in the Hindi language (LSA-H) and evaluated its psychometric properties. The scale demonstrated validity and reliability as a comprehensive tool for assessing mobility among community-dwelling older adults. Mobility is one of the important components in the life cycle of older adults to preserve their autonomy and independence. LSA scores were related not only to the mobility limitations but also to the physical performance and disability of older adults.

India, characterized by its multiethnic and multicultural composition, presents a heterogeneous sociodemographic landscape that extends across state boundaries. The LSA scale’s cross-cultural validity and utility receive robust support from participants hailing from four Hindi-speaking states, representing diverse geographical regions within India. The multicentric design enhances the scale’s applicability and relevance to a wide array of populations. Furthermore, the balanced enrollment across various centers enhances representativeness and mitigates selection bias. This methodological approach strengthens the scale’s validity and generalizability, making it a valuable tool for assessing life space mobility across diverse Indian populations.

The mean LSA-H score in the present study was 56.53, which is comparable to that reported in earlier studies from different countries, while it was much lower in the Turkish version [46–48]. This difference may be explained by the fact that the respondents in the Turkish version of the LSA were older than those in our study were, suggesting that life space mobility decreases with increasing age. Previous studies have also mentioned the negative correlation between life space mobility and increasing age among older adults [49, 50].

The present study assessed item-level scale-level content validity indices, which were 0.967 and 0.965, respectively, which were greater than those of the Chinese and Cantonese versions, indicating that the concept of life space was well represented in all the items [51]. The correlations between the LSA-H score and the ABC-H score and between the physical health subscale score and the WHO BREFF score were 0.707 and 0.706, respectively, revealing a strong correlation between physical function and life space mobility and confirming the validity of the criterion. Previous studies in different countries have shown that the translation of the LSA is positively correlated with various scales used to assess physical function; these scales include the Multidimensional Functional Assessment Questionnaire (MFAQ), the General Health subscale of the Short-Form-36 Health Survey (GH of the SF-36), the Physical Activity Scale for the Elderly (PASE), and the Short Physical Performance Battery (SPPB) [20, 21, 46].

In this study, the LSA-H score was negatively correlated with depression, with a correlation coefficient of – 0.674 amongst older adults. Similar findings were observed in the study conducted by Tseng et al. for validation of the Chinese version of the LSA using the 10-item Center for Epidemiological Studies Depression Scale (CESD-10), for which the correlation coefficient was − 0.54 [21]. Studies conducted by Gyasi RM et al. and Musich S et al. showed that mobility limitations can lead to significant depressive symptoms among older adults [52, 53].

The composite interclass coefficients of the Hindi version of the LSA showed excellent reliability for level 1 restraint but the lowest reliability for level 5 restraint. In the present study, the test-retest reliability assessment showed good reliability for all individual subscores as well as for all composite scores, similar to the findings of the study conducted by Ho LYW et al. [51]. The lowest Intraclass Correlation Coefficient (ICC) sub-score is observed for level 3 (0.738) compared to other sub-scores of life space. This discrepancy may be attributed to the circumstances surrounding the data collection process, particularly during the COVID-19 pandemic lockdown period. It is plausible that during this time, restrictions on mobility outside the home directly impacted the assessment of level 3 and above scores, leading to lower reliability in these categories. Such contextual factors should be considered when interpreting the ICC values and their implications for the assessment of life space mobility.

The Standard Error of Measurement (SEM) serves as a metric for assessing the extent to which variation in scores reflects true change. In the present study, the SEM and SEM% for the life space composite score were calculated as 6.107 and 5.08%, respectively. A study by Kammerlind et al. reported SEM and SEM% values for the life space level composite score as 9.1 points (7.5%), while another study by Simões M do SM et al. reported values of 4.12 (3%), respectively [47, 54]. Notably, no ceiling or floor effects were observed in the composite LSA Hindi version questionnaire. The observed SEM% falls within an acceptable range, especially considering that the interval between measures was only one week, which may have minimized vulnerability to recall bias among participants. Additionally, upon analyzing life space sub-score levels, SEM% values were found to be less than 10% for life space level 1, level 2, and level 5 sub-scores. However, for life space level 3 and level 4 sub-scores, SEM% fell between 10 and 20%. The variability in the life space level 4 and 5 sub-scores could potentially be attributed to movement restrictions during the lockdown period in certain areas due to the COVID-19 pandemic, as these sub-scores are based on movements outside the neighborhood area and beyond the village or town. Nonetheless, SEM% values for all level sub-scores remained within acceptable ranges. It’s worth noting that no other study with information on the assessment of life space level sub-score analysis was identified. This highlights the novelty and significance of the present study’s findings in evaluating life space mobility at various levels within the Hindi-speaking population.

In this study, the LSA-H questionnaire demonstrated adequate internal consistency, indicating the extent to which the questionnaire’s items are interrelated and measure the same construct. The internal consistency, as measured by Cronbach’s alpha, was found to be satisfactory with a value of 0.924 (95% CI: 0.912–0.928). A study conducted by Garcia IFF et al. among Brazilian older adults with chronic obstructive pulmonary diseases reported a Cronbach’s alpha of 0.80 (95% CI: 0.76–0.80) [25]. Similarly, another study by Simões M do SM et al. among Brazilian community-dwelling older adults reported a Cronbach’s alpha of 0.92 (95% CI: 0.91–0.92) [47]. No other studies providing information on the calculation of internal consistency were identified. These findings underscore the reliability and consistency of the LSA-H questionnaire across different populations, further supporting its utility as a valid instrument for assessing life space mobility among Hindi-speaking individuals.

### Strengths and limitations

The findings of the present study showed that the instrument had satisfactory validity and reliability. Cross-cultural adaptation during the translation process was well accepted by the older adults in the Hindi version. The instrument assesses mobility function in a short duration of just 3 to 5 min. The instrument can be used for community-level screening for mobility as well as in research for quick assessment of functional mobility. This study has several limitations. The present study did not assess sex differences in LSA-H scores. As in the Indian scenario, the majority of the females limited their activity to various household works, while males preferred to be involved in out-of-home activities. Therefore, females usually have less life space than males. During the translation process, the exact distance to a neighborhood or outside the home was not mentioned, which can lead to different interpretations by individuals. During the study, geographical locations, such as rural, urban, and tribal/hilly areas, were not considered because a lack of transportation facilities in a particular area can alter the scores. The availability of personal assistance to caregivers in the house was not assessed, which would have a direct impact on the life space environment. Finally, the study’s criterion-related validity was examined against subjective indicators (ABC and WHO-BREF) where the Hindi version of the instruments was available. However, since the LSA accurately reflects mobility accurately, assessing its relationship with objective measures of walking ability, and physical activity levels from the life space perspective, is necessary. This issue represents a significant limitation of the current study. The instrument is questionnaire-based and subjective, while objective metrics are more valid and reliable.

## Conclusion

This study demonstrated that the Hindi version of the LSA is a valid and reliable instrument for assessing living space among older adults in the Hindi language in the Indian population. Furthermore, the LSA-H was significantly correlated with other health assessment tools in terms of functional mobility, general health status and mental well-being. The LSA-H can be used as a tool for quick assessment by clinicians and can be used to guide community interventions for active aging.

## Data Availability

The datasets used and/or analyzed during the current study are available from the corresponding author upon reasonable request.

## Abbreviations

ABC- H: Activities-Specific Balance Confidence Scale Hindi
ADL: activities of daily living
AIIMS: All India Institute of Medical Sciences
CESD: Center for Epidemiological Studies Depression Scale
CI: Confidence Interval
CVI: Content validity index
EMS: Elderly Mobility Scale
FSQ: functional status questionnaire
GDS-SFH: Geriatric Depression Scale: Short Form Hindi
HABAM: Hierarchical Assessment of Balance and Mobility
H-MMSE: Hindi version of the Mini-Mental State Questionnaire (H-MMSE)
IADL: instrumental activities of daily living
ICC: Intraclass correlation coefficient
I-CVI: item-level content validity index
INI: Institutes of National Importance
ISC-08: International Standard Classification of Occupations
IQR: Interquartile range
LSA: Life-Space Assessment
LSA- H: Life-Space Assessment (Hindi Version)
Lower LOA: Lower level of agreement
MMFQ: Multidimensional Functional Assessment Questionnaire
PASE: Physical Activity Scale for the Elderly
PPME: Physical Performance Mobility Examination
S-CVI: scale level content validity index
SD: Standard Deviation
SEM: Standard error of measuremnt
SF-36: 36-Item Short Form Survey
SPPB: Short Physical Performance Battery
UAB: University of Alabama at Birmingham
Upper LOA: Upper level of Agreement
